# THE BENEFITS OF CREATIVE ARTS INTERVENTIONS FOR OLDER ADULTS: THE SOCIOCULTURAL MODEL AND THE DEMENTIA STUDIES MODEL

**DOI:** 10.1093/geroni/igae098.2237

**Published:** 2024-12-31

**Authors:** Carolyn Adams-Price

**Affiliations:** Mississippi State University, Mississippi State, Mississippi, United States

## Abstract

Over the past 25 years, there has been a huge increase in the employment of interventions for older adults that incorporate as a primary feature participation in creative activities. Interventions have focused on art domains as distinct as photography, dance, music, traditional handicrafts (such as quilting or pottery), and many others. In addition, programs have been created for older adults with a huge range of ability levels and cognitive efficacy, from highly functioning older adults to older adults with severe cognitive deficits. The purpose of this paper will be to describe the benefits of creative interventions for different types of older adults with different types of capabilities. Two different perspectives will be highlighted; including the sociocultural model of creativity, which is linked to life-span developmental psychology, and the dementia studies model, which derives from holistic and qualitative approaches to aging. The paper will discuss the ways in which these two approaches are fundamentally different, and the ways in which they are similar, and discuss what they have to say about the nature of creativity.

